# Involvement and regulation of the left anterior cingulate cortex in the ultrasonic communication deficits of autistic mice

**DOI:** 10.3389/fnbeh.2024.1387447

**Published:** 2024-05-15

**Authors:** Yilin Hou, Yuqian Li, Dingding Yang, Youyi Zhao, Tingwei Feng, Wei’an Zheng, Panpan Xian, Xufeng Liu, Shengxi Wu, Yazhou Wang

**Affiliations:** ^1^Department of Military Medical Psychology, Fourth Military Medical University, Xi’an, China; ^2^Department of Neurobiology and Institute of Neurosciences, School of Basic Medicine, Fourth Military Medical University, Xi’an, China; ^3^State Key Laboratory of Military Stomatology & National Clinical Research Center for Oral Diseases & Shaanxi Engineering Research, Center for Dental Materials and Advanced Manufacture, Department of Anesthesiology, School of Stomatology, Fourth Military Medical University, Xi’an, China

**Keywords:** autism spectrum disorder, anterior cingulate cortex, ultrasonic vocalization, fragile X mental retardation 1, valproic acid, precise target transcranial magnetic stimulation

## Abstract

**Introduction:**

Autism spectrum disorder (ASD) is a group of diseases often characterized by poor sociability and challenges in social communication. The anterior cingulate cortex (ACC) is a core brain region for social function. Whether it contributes to the defects of social communication in ASD and whether it could be physiologically modulated to improve social communication have been poorly investigated. This study is aimed at addressing these questions.

**Methods:**

Fragile X mental retardation 1 (FMR1) mutant and valproic acid (VPA)-induced ASD mice were used. Male–female social interaction was adopted to elicit ultrasonic vocalization (USV). Immunohistochemistry was used to evaluate USV-activated neurons. Optogenetic and precise target transcranial magnetic stimulation (TMS) were utilized to modulate anterior cingulate cortex (ACC) neuronal activity.

**Results:**

In wild-type (WT) mice, USV elicited rapid expression of c-Fos in the excitatory neurons of the left but not the right ACC. Optogenetic inhibition of the left ACC neurons in WT mice effectively suppressed social-induced USV. In *FMR1^−/−^*- and VPA-induced ASD mice, significantly fewer c-Fos/CaMKII-positive neurons were observed in the left ACC following USV compared to the control. Optogenetic activation of the left ACC neurons in *FMR1^−/−^* or VPA-pretreated mice significantly increased social activity elicited by USV. Furthermore, precisely stimulating neuronal activity in the left ACC, but not the right ACC, by repeated TMS effectively rescued the USV emission in these ASD mice.

**Discussion:**

The excitatory neurons in the left ACC are responsive to socially elicited USV. Their silence mediates the deficiency of social communication in *FMR1^−/−^* and VPA-induced ASD mice. Precisely modulating the left ACC neuronal activity by repeated TMS can promote the social communication in *FMR1^−/−^* and VPA-pretreated mice.

## Introduction

Autism spectrum disorder (ASD) is a group of developmental disorders that are behaviorally diagnosed and characterized by deficits in social communication and restricted, repetitive behaviors or interests that affect approximately 1% of children around the world (Lord et al., 2018; Dai et al., 2022; Lundstrom et al., 2022). Deficits in social communication are a prominent syndrome in ASD children, showing impairment of pragmatic language and unwillingness to communicate (Silleresi et al., 2020; Reindal et al., 2023), for which the underlying mechanisms remain largely unknown, and effective treatments are still lacking.

Multiple ASD mouse models, both genetic factors and environmental factors induced, have been established (Pietropaolo et al., 2017; Schroeder et al., 2017). The majority exhibited defects in ultrasonic vocalization (USV), an equivalent of human speech (Takumi et al., 2020). For example, mutations in the fragile X messenger ribonucleoprotein 1 (*FMR1*) gene, the most common risk gene for ASD, lead to severe USV defects (Hodges et al., 2017). Prenatal exposure to valproic acid (VPA, the most widely adopted environmental factor to induce ASD) results in a reduction of USV emissions (Tsuji et al., 2021). As mouse USV is closely associated with social activity and conveys both information and emotion (Lahvis et al., 2011), investigating the underlying mechanisms of USV defects in ASD mouse models would give insight into social communication in human patients (Sungur et al., 2016).

In general, USV emission is thought to be mediated by a direct projection from the motor cortex to vocal motoneurons in the brainstem and an indirect cortico-striatal-thalamic circuit (Arriaga and Jarvis, 2013; Tschida et al., 2019). Given that USV is usually elicited by social activity, it is highly possible that social brain regions are involved in USV production or regulation, which, however, has been poorly investigated. The anterior cingulate cortex (ACC) is a core brain region integrating multiple social information (Tervo et al., 2014) and mediating social cognition, social decision, and social reward (Schneider et al., 2020). Dysfunction of synaptic transmission in the ACC underlies social dysfunction in multiple ASD mouse models (Guo et al., 2019; Block et al., 2022), so it is interesting to explore whether it is involved in ASD-associated USV deficiency.

To modulate neuronal activities in a given brain region, we adopted the optogenetic technique and repeated transcranial magnetic stimulation (rTMS), two powerful neuronal modulation methods that have made great progress in the past decade. Optogenetic manipulation starts with artificially expressing photosensitive opsins (e.g., ChRs and NpHR) in the target brain region or neurons and is achieved by photostimulation of these opsins (Kim et al., 2017). Light activation of ChRs is used to activate neurons, while light activation of NpHR is used to inhibit neurons. rTMS is a promising non-invasive brain-stimulating method that induces electronic currents in the brain by pulsating magnetic fields. It has been clinically applied to many mood-defective disorders (Camacho-Conde et al., 2022). Previous animal studies, including ours, have demonstrated its beneficial effects on social interaction (Hou et al., 2021). However, most of the rTMS used in animal studies covers the whole cerebral cortex, making the results difficult to explain. In the present study, by adopting the optogenetic technique, we investigated the role of the ACC neurons in ASD-associated USV deficits, and by applying a newly developed 3-coil TMS transducer that could confine the magnetic field specifically over the ACC, we evaluated its effects on social communication.

## Methods

### Mice

Adult C57 mice and FVB129 mice were bought from the animal center of the Fourth Military Medical University. *FMR1^−/−^* mice were bought from Jax laboratory (stock No.010504). All animal experiments were carried out under protocols approved by the Animal Care and Use Committee of Fourth Military Medical University and according to “Policies on the Use of Animals and Humans in Neuroscience Research,” revised and approved by the Society for Neuroscience.

VPA-treated mice were obtained by breeding the progenies of pregnant mice exposed to VPA at E12.5 (i.p. injection, 500 mg/kg, P4543, Sigma).

### Immunohistochemistry

For immunohistochemistry, mice were perfused intracardially with 4% paraformaldehyde phosphate buffer. Serial coronal sections were prepared and blocked by PBS containing 3% BSA and 0.3% Triton-X100, and then incubated with primary antibodies overnight at room temperature. The primary antibodies used were as follows: mouse anti-CaMK II (Cell Signaling, 50,049, RRID: AB_2721906, 1: 400), mouse anti-GAD67 (Millipore, MAB5406, RRID: AB_2278725, 1:1000). mouse anti-NeuN (Abcam, ab104224, RRID: AB_10711040, 1:200), and rabbit anti-c-Fos (cell signaling, 2,250, RRID: AB_2247211, 1:1600). After washing with PBS, corresponding secondary antibodies conjugated with donkey anti-rabbit (Alexa Fluor 488, Invitrogen, A-21206, RRID: AB_2535792, 1:800), and donkey anti-mouse (Alexa Fluor 594, Invitrogen, A-21203, RRID: AB_141633, 1:800) were incubated with the sections for 2–4 h at room temperature, protected from light. After washing with PBS, sections were counterstained with Hoechst33342 (1:1000, Sigma) for 20 min.

### Virus injection and optogenetic modulation

rAAVhSyn-hChR2-mCherry (serotype 2/9, titer 4 × 10^12^ vg/ml) and rAAVhSyn-eNpHR3.0-mCherry (serotype 2/9, titer 4 × 10^12^ vg/ml) were purchased from Brain VTA, Wuhan. For virus injection, the animals were anesthetized with isoflurane. The injections were made into the left ACC (0.23 mm left to bregma, 0.93 mm anterior to bregma, and 1.6 mm in depth). Standard injection volumes were set to 200 nL. After the injection, the needle was left in place for 10 min before being withdrawn.

For optogenetic neuronal modulation, the ACC was activated with a 465-nm laser (49 mW, 10 Hz, blue light) for 30 s for hChR2 and inhibited with a 589-nm laser (49 mW, 10 Hz, yellow light) for 30 s for eNpHR 3.0.

### Ultrasonic vocalization recording

USV recordings were performed as described (Hou et al., 2021). Briefly, mice were placed in a clean rectangular cage (60 × 42 × 40 cm) covered with a metal lid. A field microphone (VT UltraMic-384, Virtins Technology) was placed 2 cm below the lid of the cage. The microphone signal was sampled by software (SeaWave), recording frequencies ranging from 20 Hz to 190 kHz. One male experimental mouse and one female WT mouse were put in the USV recording cage. Each group of mice had no interactions prior to the recording and was considered as *n* = 1 for statistics. USV production was recorded for 10 min. The data were stored and analyzed offline by a researcher blinded to the experimental design. USV was detected using a custom program (deep squeak) with a spectrographic display.

### Brain region specificity test of precise target TMS

A newly developed TMS device equipped with a 13-layer 3-turn “8”-shape coil, capable of focusing the stimulation region within a minimal size of 0.5 mm^3^ (Black Dolphin IT-TMS, Solide Company, Xi’an, China), was adopted. Before formal experiments, the brain region specificity was tested by stimulating the hindlimb region of the right motor cortex. Upon single TMS stimulation, the movement of bilateral hindlimbs was videoed. At the same time, electromyographic activity in the bilateral hindlimbs was recorded as described (Saturnino et al., 2020). Briefly, the positive electrode was inserted into the front end of the gastrocnemius muscle. The negative electrode was inserted into the back end of the gastrocnemius muscle, and the grounding electrode was inserted into the root of the mouse tail. The maximum signal sampling rate was set to 32,000 Hz. The single-channel signal sampling rate was set to 8,000 Hz. The signal was filtered through a 50-Hz band-stopping filter, a 20–480-Hz band-passing filter, and a power frequency harmonic filter (Solide Company, Xi’an, China).

### Precise target rTMS treatment

rTMS was carried out by placing the probe 1–2 mm above the skull of the left ACC. The parameters of rTMS were as follows: 10 Hz stimulating pulse intensity at 40% of the maximum power of the rTMS device, 20 stimulations per cluster, repeated 40 times. rTMS was conducted for 7 consecutive days, with 800 pulses per day. Starting 3 days prior to the rTMS procedure, the mice were habituated to the coil for 10 min each day. For sham stimulation in the control group, the coil was placed immediately above the skull without magnetic stimulation.

For electric field simulation, the open-access electromagnetic simulation software SimNIBS was used as described (Saturnino et al., 2019, 2020). The coil inductance was approximately 17 μH. The capacitance was 140 μF. The reference voltage was 1,600 V, and the reference frequency was 3,250 Hz. The maximum current change rate of the coil was approximately 9.4e7 A/s. According to the TMS stimulation intensity, the current change rate was set to approximately 40% of the maximum current change rate.

### Statistical analysis

All behavior analysis and statistics were performed by an investigator who was blinded to the experimental design. No sample calculation was performed. For morphological analysis, three mice were included in each group, and at least five brain sections were quantified for each mouse. For behavior analysis, eight mice were included in each group. No mice were excluded in terms of data analysis. Mice of the same genotype were randomly assigned to the same treatment group. Each behavioral test was conducted using distinct groups of animals. Data are presented as the mean ± standard error. Normal distribution is assessed using the Shapiro–Wilk test. Statistical comparisons were made using the Student’s t-test or one-way ANOVA. A *p*-value of less than 0.05 was considered statistically significant.

## Results

### Involvement of the left ACC neurons in social-induced USV in wild-type (WT) mice

**Figure 1:**
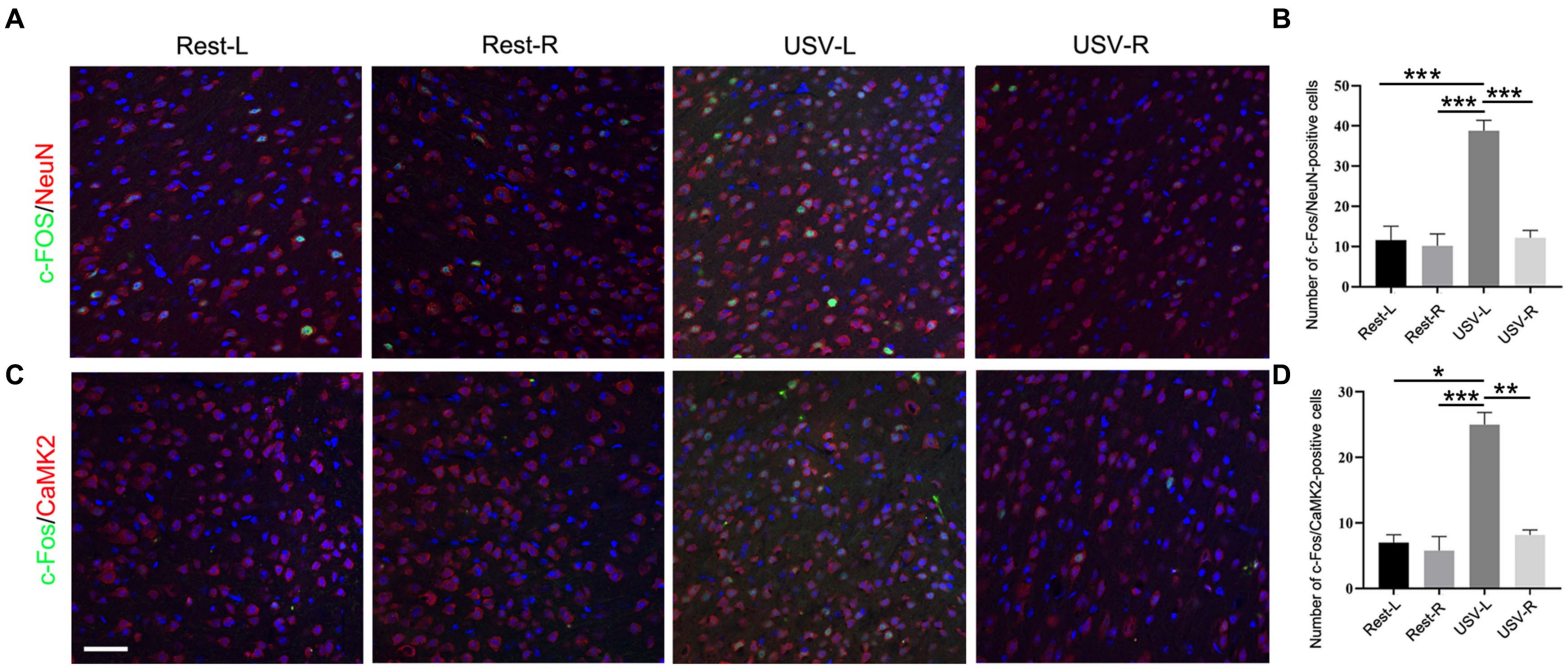
Expression of c-Fos in the left and right ACC of WT mice following USV emission. **(A,B)** Double immunostaining and quantification of c-Fos/NeuN in the left and right ACC under the resting condition or following USV emission. **(C,D)** Double immunostaining and quantification of c-Fos/CaMKII in the left and right ACC under the resting condition or following USV emission. Notice the induction of c-Fos/NeuN- and c-Fos/CaMKII-positive cells in the left ACC by USV. One-way ANOVA in **(B,D)**, *N* = 3 mice per group; *p < 0.05; **p < 0.01; ***p < 0.001; Bar= 80 μm; L, left; R, right.

To examine if the ACC neurons respond to social-induced USV, we put a male mouse and a female mouse together for 10 min to induce USV and conducted double-immunostaining of c-Fos with pan-neuronal marker NeuN, excitatory neuronal marker CaMKII, and inhibitory neuronal marker GAD67 in the bilateral ACC following USV emission. The results showed a significantly larger number of c-Fos/NeuN-positive cells in the left ACC of the USV-emitting mice compared to the right ACC of the USV-emitting mice and that of the bilateral ACC of the resting mice (one-way ANOVA). *P*_*Rest-L* vs. *USV-L*_ = 0.0002. *P*_*Rest-R* vs. *USV-L*_ = 0.0007. *P*_*USV-L* vs. *USV-R*_ = 0.0004 (Figures 1A,B). Similarly, a significant increase in the c-Fos/CaMKII-positive cells was observed in the left ACC of USV-emitting mice, compared to the right ACC of the USV-emitting mice and the bilateral ACC of the resting mice (one-way ANOVA). *P*_*Rest-L* vs. *USV-L*_ = 0.011. *P*_*Rest-R* vs. *USV-L*_ = 0.0002. *P*_*USV-L* vs. *USV-R*_ = 0.001 (Figures 1C,D). No significant difference in the c-Fos/NeuN- and c-Fos/CaMKII-positive cells was found between the bilateral ACC of the resting mice and the right ACC of USV-emitting mice (Figures 1B,D). Co-expression of c-Fos with GAD67 was rarely detected in the bilateral ACC of both USV-emitting mice and resting mice (Supplementary Figure S1). These data demonstrated that the left ACC was highly responsive to social-induced USV.

**Figure 2:**
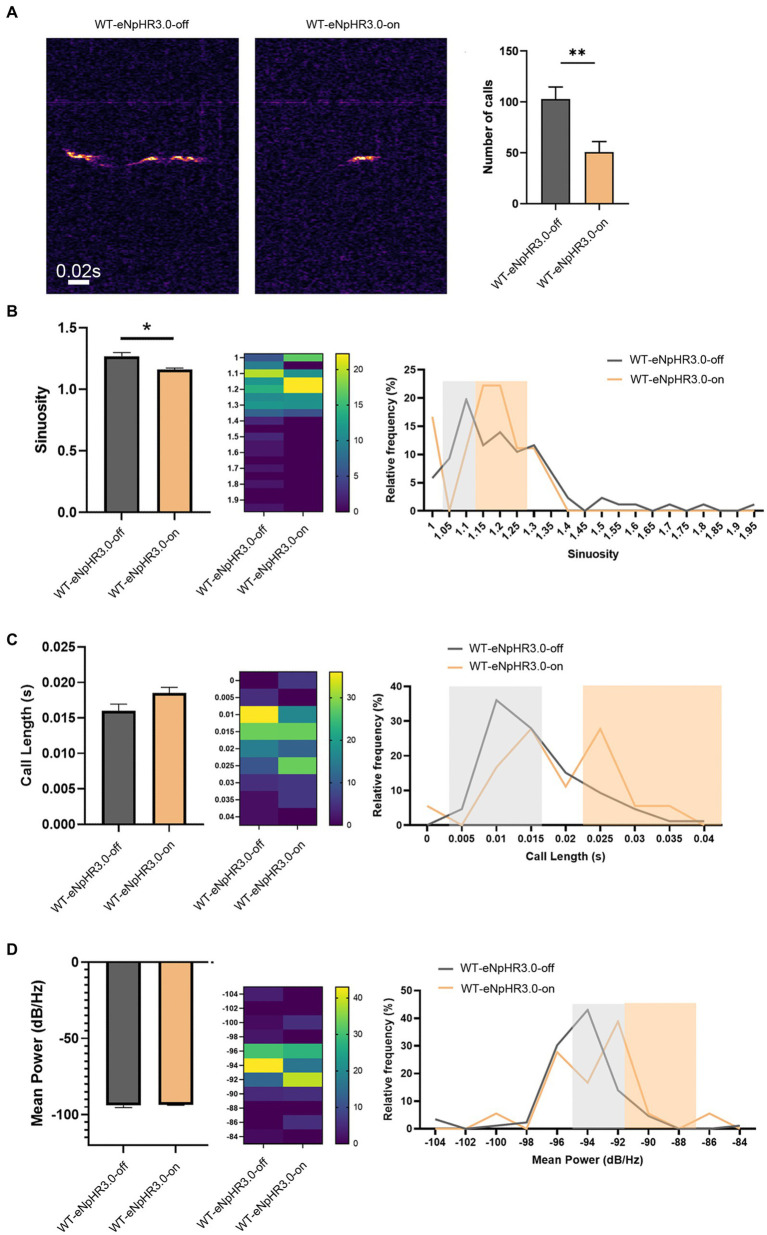
Effects of optogenetic inhibition of the left ACC on socially induced USV. **(A)** USV emission in WT mice before laser irradiation (WT-eNpHR3.0-off) or after laser irradiation (WT-eNpHR3.0-on). Notice the decrease in call numbers following optogenetic inhibition. **(B)** Quantification and distribution of call sinuosity in WT mice before or after laser irradiation. **(C,D)** Quantification and distribution of call length and mean power before or after laser irradiation. Notice that optogenetic inhibition shifted a portion of calls toward longer duration or higher power. Student’s t-test in **(A–D)**, *n* = 8 mice per group; *p < 0.05; **p < 0.01.

To examine whether the left ACC was required for social-induced USV, we carried out optogenetic modulation of the ACC neurons. AAV expressing light-sensitive eNpHR3.0 was injected into the left ACC of WT mice. The ACC injection and virus infection were verified by mCherry expression (Supplementary Figure S2). Optogenetic inhibition of the ACC neurons was achieved by laser irradiation at 589 nm for 30 s, and USV was evaluated immediately within the following 10 min. The results showed that the optogenetic inhibition of the left ACC significantly reduced the numbers (Student’s *t*-test. *p* = 0.0078, Figure 2A), as well as the sinuosity of USV emitted (Student’s *t*-test. *p* = 0.029, Figure 2B). Although no change in average call length or mean power of USV was found before or after laser irradiation, suppressing neuronal activity seemed to shift calls toward longer duration and higher power (Figures 2C,D). These data indicated that the left ACC neurons may play a role in social communication.

### Loss of the left ACC neuronal responsiveness to social-induced USV in *FMR1^−/−^* and VPA-pretreated mice

**Figure 3:**
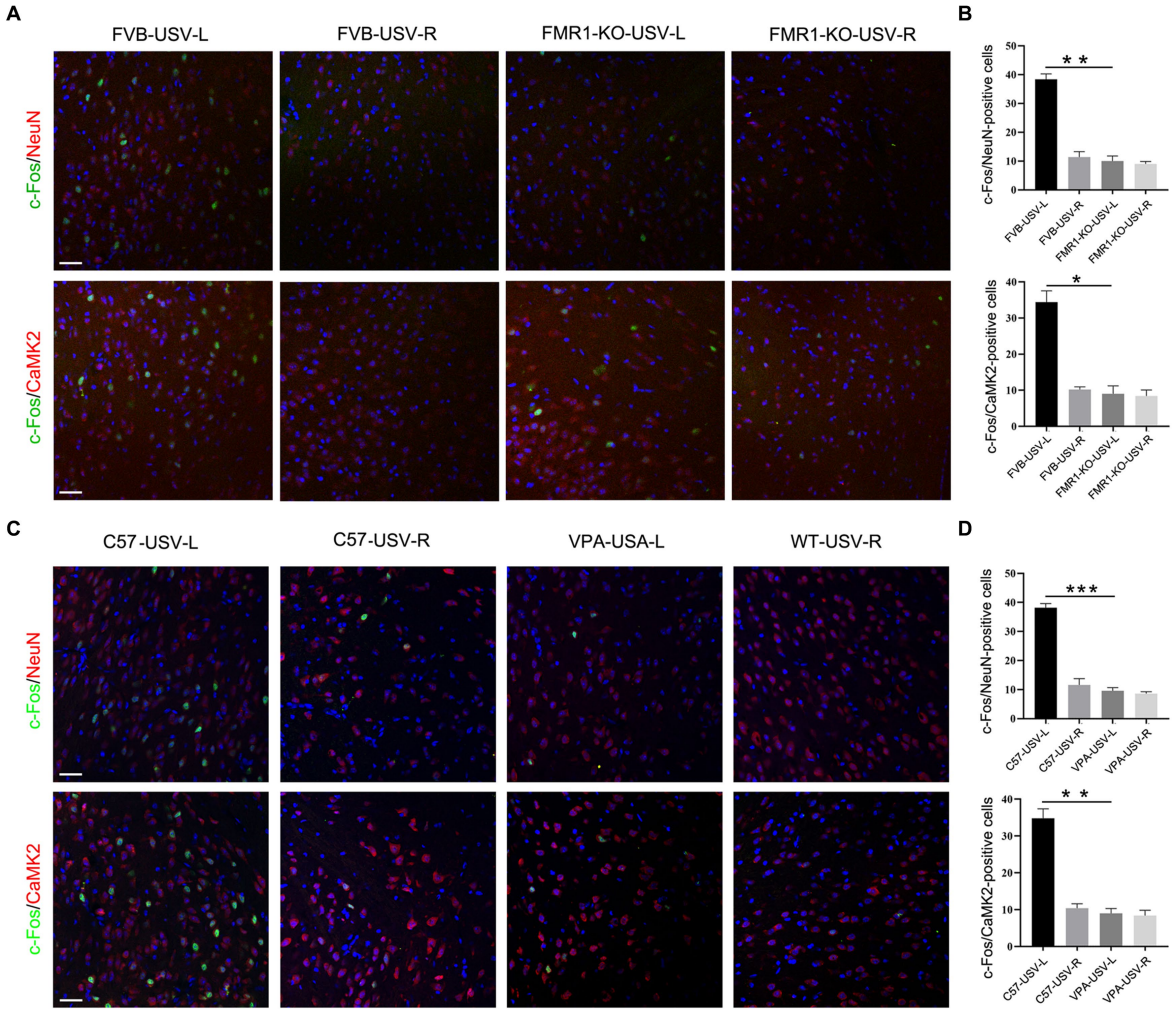
Expression of c-Fos in the left and right ACC of *FMR1^−/−^* and VPA-pretreated mice following USV emission. **(A,B)** Double-immunostaining and quantification of c-Fos/NeuN and c-Fos/CaMKII in the left and right ACC of *FMR1^−/−^* mice following USV emission. Notice the abolishment of c-Fos expression in the left ACC of *FMR1^−/−^* mice. **(C,D)** Double-immunostaining and quantification of c-Fos/NeuN and c-Fos/CaMKII in the left and right ACC of VPA-pretreated mice following USV emission. Notice the reduction of c-Fos expression in the left ACC of VPA-pretreated mice. One-way ANOVA in **(B,D)**, *n* = 3 mice per group. *p < 0.05; **p < 0.01; ***p < 0.001; Bars = 80 μm; L, left; R, right.

We next tested whether the left ACC was involved in the social communication deficits in *FMR1^−/−^* mice and VPA-pretreated mice. Considering that *FMR1^−/−^* mice were derived from the FVB129 background, we used FVB129 mice as a WT control for *FMR1^−/−^* mice. USV induced a large number of c-Fos/NeuN- and c-Fos/CamKII-positive cells in the left ACC of FVB129 mice. Significant reductions of c-Fos/NeuN- and c-Fos/CamKII-positive cells were observed in the left ACC of *FMR1^−/−^* mice, compared to those in FVB129 control mice (one-way ANOVA, *P*_*FVB-USV-L* vs. *FMR1-KO-USV-L*_ = 0.002 for c-Fos/NeuN-positive cells). *P*_*FVB-USV-L* vs. *FMR1-KO-USV-L*_ = 0.02 for c-Fos/CamKII-positive cells (Figures 3A,B). USV did not induce a c-Fos response in the right ACC of both *FMR1^−/−^* mice and FVB129 control (Figures 3A,B).

In VPA-induced ASD mice, a smaller number of c-Fos/NeuN- and c-Fos/CamKII-positive cells were found in the left ACC following USV compared to the left ACC of the control mice (one-way ANOVA, *P*_*C57-USV-L* vs. *VPA-USV-L*_ = 0.0003 for c-Fos/NeuN-positive cells). *P*_*C57-USV-L* vs. *VPA-USV-L*_ = 0.001 for c-Fos/CamKII-positive cells (Figures 3C,D). Few c-Fos-positive cells were detected in the right ACC of both C57 mice and VPA-pretreated mice (Figures 3C,D). These data demonstrated a weak or absent response of the left ACC in ASD mice to USV.

### Optogenetic activation of the left ACC neuronal activity increases USV in *FMR1^−/−^* and VPA-pretreated mice

**Figure 4:**
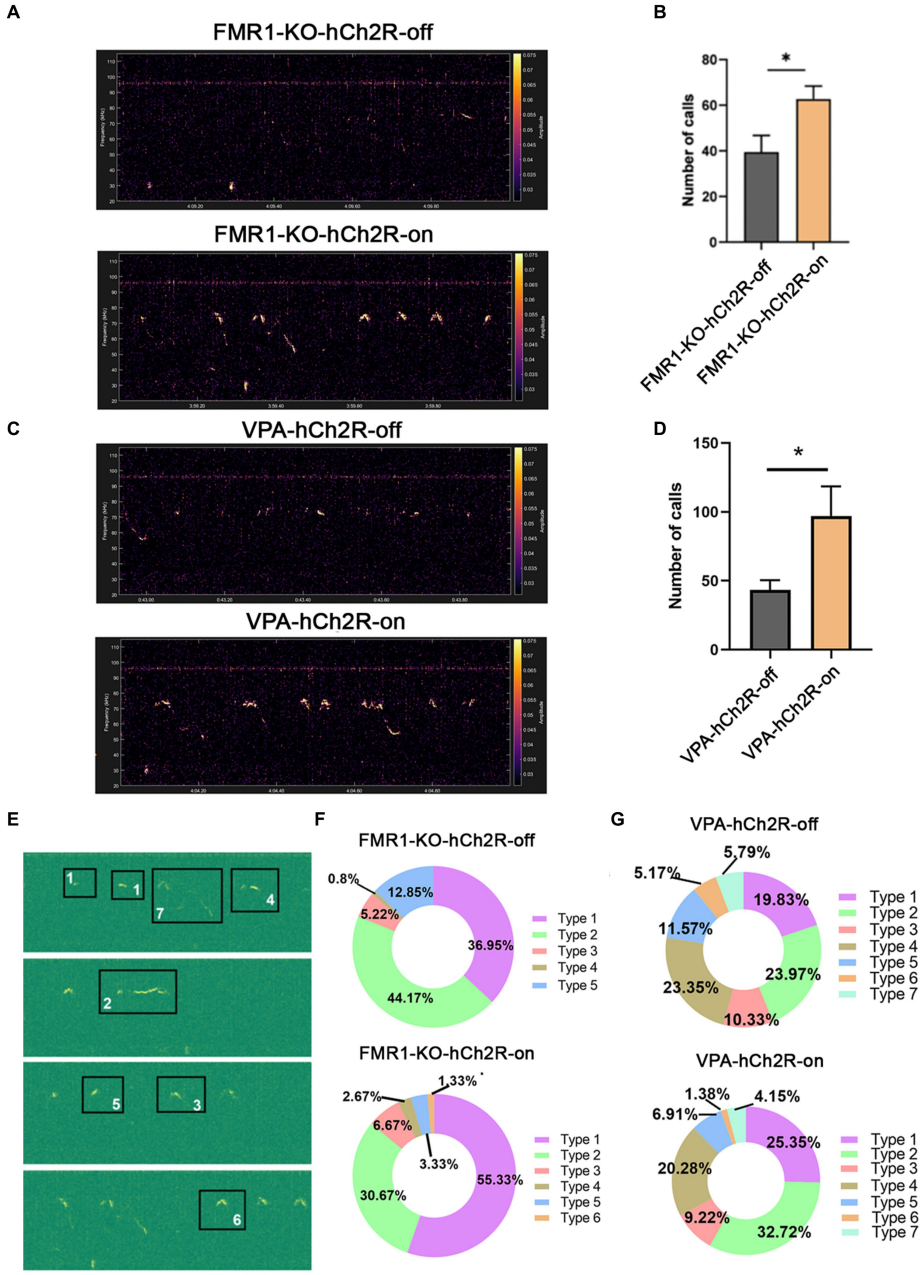
Effects of optogenetic activation of the left ACC on socially induced USV in *FMR1^−/−^* and VPA-pretreated mice. **(A,B)** Representative tracing and quantification of USV calls in *FMR1^−/−^* mice before (FMR1-KO-hCh2R-off) or after optogenetic activation (FMR1-KO-hCh2R-on). **(C,D)** Representative tracing and quantification of USV calls in VPA-pretreated mice before (VPA-KO-hCh2R-off) or after optogenetic activation (VPA-KO-hCh2R-on). Notice the increase of USV calls in both mice due to the activation of left ACC neurons. **(E)** Definition of the subtypes of USV calls. “1” short; “2” flat; “3” split; “4” downward; “5” upward; “6” chevron; “7” complex. **(F)** USV syllables of *FMR1^−/−^* mice before or after optogenetic modulation. **(G)** USV syllables of VPA-pretreated mice before or after optogenetic modulation. Notice the increase of the type 1 syllables in both mice after the optogenetic activation of the left ACC. Student’s *t*-test in **(B,D)**, *n* = 8 mice per group; *p < 0.05.

We then evaluated the effects of increasing neuronal activity in the left ACC on social communication in ASD mice. AAV expressing hChR2 was injected into the left ACC of these two mice. Two weeks later, optogenetic activation of the ACC neurons was achieved by laser irradiation at 453 nm for 30 s, and USV was evaluated immediately within the following 10 min. Laser irradiation significantly increased the number of USV calls in both *FMR1^−/−^* and VPA-pretreated mice (Student’s *t*-test. *p* = 0.016 for *FMR1^−/−^* mice. *p* = 0.046 for VPA-pretreated mice Figures 4A–D). No changes in USV duration and frequency were detected in both mice before and after optogenetic activation (Supplementary Figure S3).

It is thought that the syllable composition of USV contains biological meanings (Von Merten et al., 2014; Chen et al., 2021). Next, we classified the syllables into seven types as described with modification (Gao et al., 2019): (1) short, a duration <10 ms; (2) flat, a consistent duration >10 ms; (3) split, an emission with a very short split; (4) downward; (5) upward; (6) chevron, a continuous increase followed by a continuous decrease in frequency; (7) complex, long duration with variation of frequency. The results showed that *FMR1^−/−^* mice exhibited five types of syllables. Activating the left ACC neurons resulted in six types of syllables and increased the percentage of type 1 syllables. VPA-pretreated mice showed all seven types of syllables under normal conditions. Optogenetic activation of the left ACC neurons did not generate more types of syllables in the VPA-pretreated mice but increased the percentage of type 1 syllables. These data indicate that activating the left ACC neurons could rescue the USV defects of *FMR1^−/−^* and VPA-pretreated mice.

### Precise target rTMS modulation of the left ACC enhances USV in WT mice

**Figure 5:**
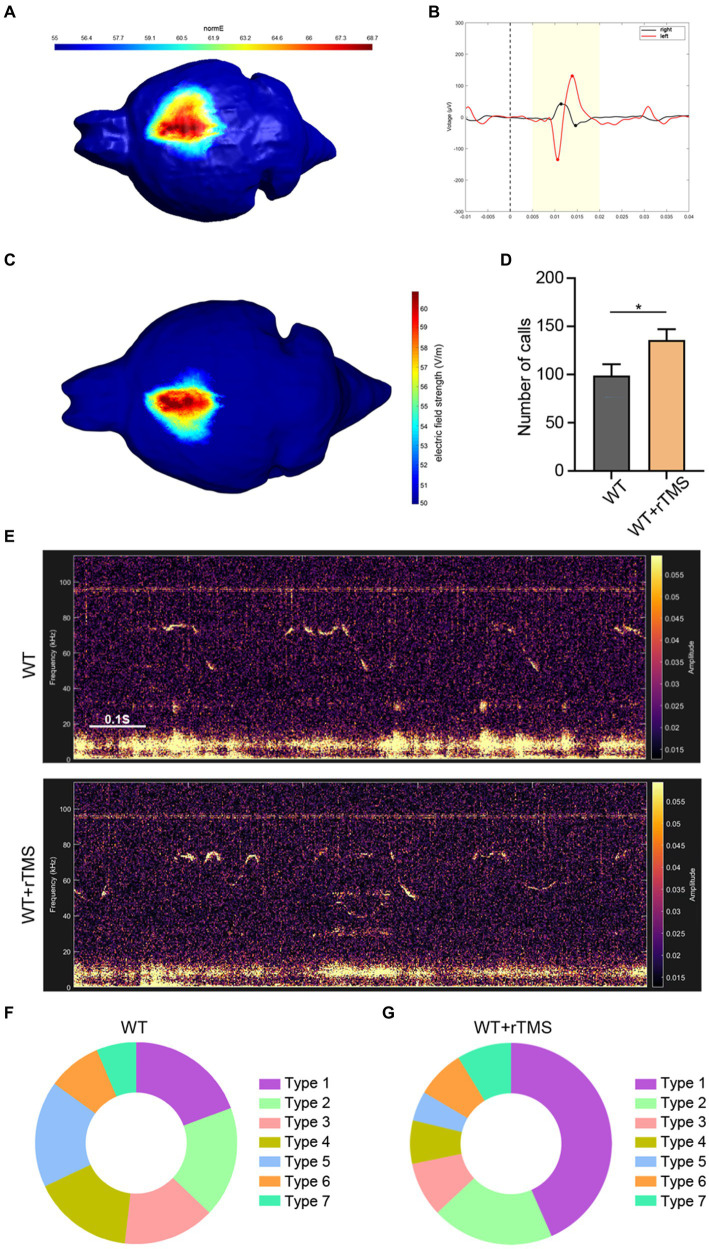
Brain region specificity of precise target rTMS and its effects of stimulating the left ACC in WT mice. **(A,B)** Brain region specificity test of rTMS by stimulating the right motor cortex **(A)** and recording electromyographic activity in bilateral hindlimbs **(B)**. Notice the movement evoked potential in the left hindlimb, but not in the right hindlimb upon TMS stimulation. **(C)** rTMS-induced electric field on the left ACC. **(D,E)** Representative tracing and quantification of USV calls in rTMS-treated WT mice. **(F,G)** USV syllables of WT mice with or without rTMS treatment. Notice the increase in total calls and type 1 syllables. Student’s *t*-test in A, *n* = 8 mice per group, *p < 0.05.

To modulate the activity of the left ACC neurons in a clinically appliable way, we developed a precise target TMS, which could confine magnetic stimulation to a size of approximately 0.5–1.5mm^3^. We first tested the region-specificity of this precise target TMS by placing the coil on the surface of the right motor cortex (Figure 5A). The left hindlimb movement was triggered immediately following TMS stimulation without affecting the movement of the right hindlimb (Supplementary Video S1). Electromyographic recording showed typical movement-evoked potential in the left hindlimb (but not in the right hindlimb) upon TMS stimulation (Figure 5B), illustrating the brain region specificity of this precise target rTMS.

Then, we stimulated the left ACC of WT mice using a 10-Hz protocol, which presumably activated the neuronal activity for 7 consecutive days and assessed USV emission (Figure 5C). Within 10 min of recording, significantly more USV calls were detected in the rTMS-treated mice (Student’s *t*-test. *p* = 0.048. Figures 5D,E). As for the syllable subgroups, the rTMS treatment did not elicit new types of USV syllables. Interestingly, type 1 syllables were more frequently observed in the rTMS-treated mice (Figures 5F,G). These data indicate that modulating the left ACC by rTMS is able to promote USV emissions.

### Precisely modulating the left ACC by rTMS partially rescues USV in *FMR1^−/−^* and VPA-induced mice

**Figure 6:**
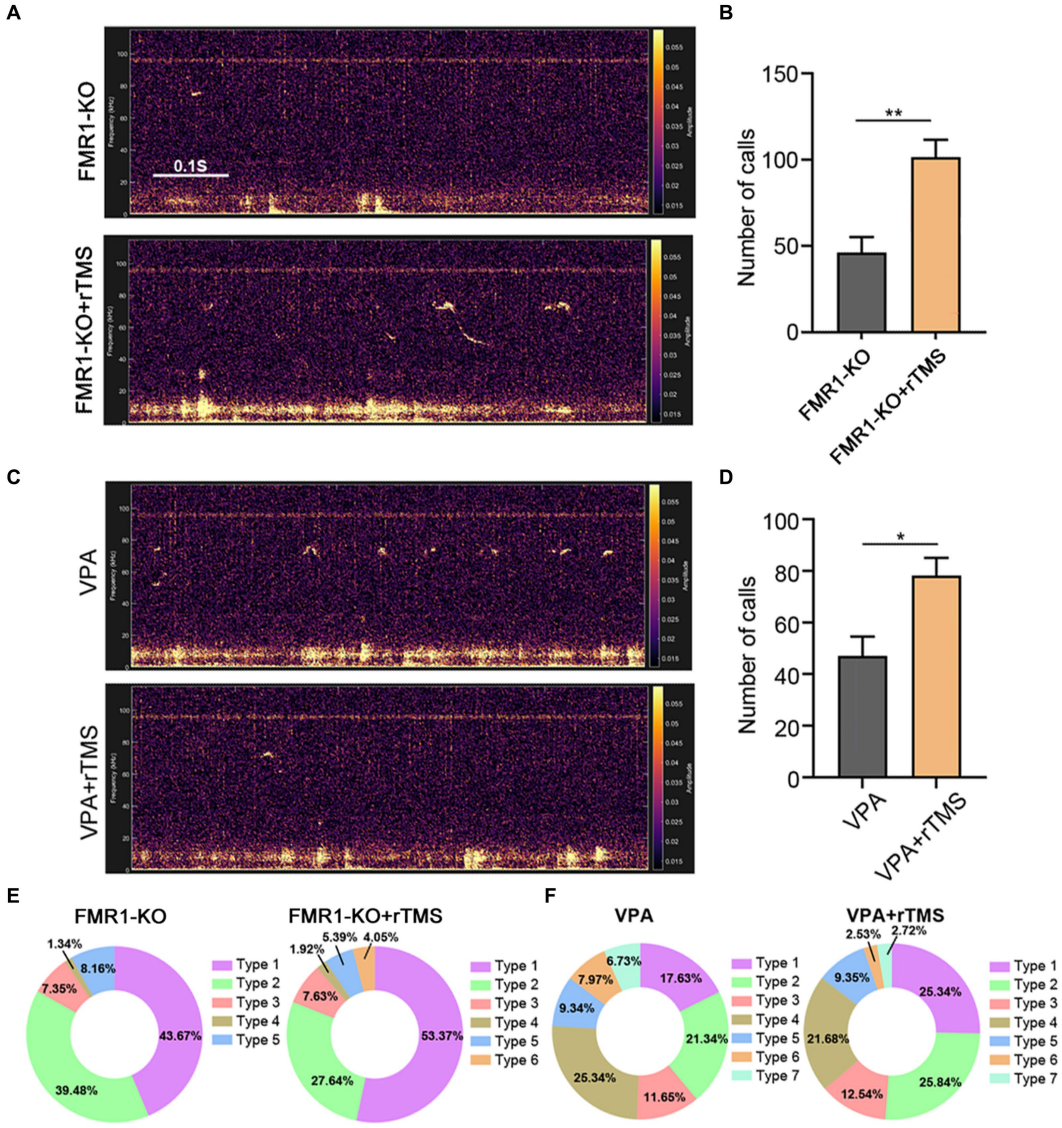
Effects of precisely modulating the left ACC by rTMS on USV emission of *FMR1^−/−^* and VPA-pretreated mice. **(A,B)** Representative tracing and quantification of USV calls in *FMR1^−/−^* mice treated with or without rTMS. **(C,D)** Representative tracing and quantification of USV calls in VPA mice treated with or without rTMS. Notice the increase in call numbers in both mice after rTMS treatment. **(E)** USV syllables of *FMR1^−/−^* mice with or without rTMS treatment. **(F)** USV syllables of VPA-pretreated mice with or without rTMS treatment. Notice the increase in type 1 syllables in both mice due to rTMS treatment. Student’s *t*-test in **(B,D)**, *n* = 8 mice per group; *p < 0.05; **p < 0.01.

We next applied the same rTMS protocol to the left ACC of *FMR1^−/−^* and VPA-pretreated mice for 7 days. A significant increase in call numbers was recorded in rTMS-treated mice, compared to sham treatment control mice (Student’s *t*-test. *p* = 0.002 for *FMR1^−/−^* mice. *p* = 0.012 for VPA-pretreated mice Figures 6A–D). To validate whether the effect was left ACC-specific, we evaluated the USV emission following rTMS stimulation of the right ACC. In this case, no significant changes in call numbers were observed (Supplementary Figure S4).

Regarding syllable composition, the rTMS treatment of the left ACC resulted in one more subtype of USV syllables in *FMR1^−/−^* mice, as did optogenetic activation. In addition, rTMS increased the percentage of type 1 syllables in both *FMR1^−/−^* and VPA-pretreated mice (Figures 6E,F), indicating that modulating the left ACC by rTMS was beneficial for social communication in ASD.

## Discussion

In the present study, by examining the expression of c-Fos in WT, *FMR1^−/−^* and VPA-induced ASD mice, we found that excitatory neurons in the left ACC respond actively to social activity-induced USV. By optogenetic modulation of the left ACC neurons in these mice, we demonstrated the requirement of the left ACC for USV emission. Furthermore, using a newly developed rTMS device that could confine the magnetic field specifically to the left ACC, we illustrated that brain region-specific modulation by rTMS could promote social communication in *FMR1^−/−^* and VPA-induced ASD mice.

At the cortical level, USV information is usually thought to be processed by the auditory cortex (Tasaka et al., 2018). As far as we know, few studies have attempted to explore whether other cortical regions were involved in USV communication. Recently, one study reported that stimulating certain regions in the ACC could elicit USVs (Gan-Or and London, 2023). Our data on optogenetic manipulation further demonstrated the crucial role of the left ACC in USV communication in mice. It is usually regarded that the left–right asymmetry of brain function is unique to humans (Corballis, 2014). Only one early study indicated that mouse USV might be left-hemisphere dominant (Ehret, 1987). Given that language is mainly encoded by the left hemisphere in humans, our data added new evidence for left hemisphere-dominated social communication in animals.

It is known that the ACC interconnects multiple brain regions to process social information (Rolls, 2019). Among these brain regions, the hippocampal neurons respond to USV (Hamed et al., 2016). In addition, there is a left–right asymmetric distribution of synapses at the hippocampal CA3-CA1 projection in mice (El-Gaby et al., 2015). It is thus possible that the left–right asymmetry of the ACC–hippocampus circuit may account for the left ACC-mediated USV, which is to be further investigated.

In ASD patients, impairment of hemispheric lateralization has been reported (Deemyad, 2022). A previous MRI study revealed poor connectivity of the ACC with the left inferior frontal gyrus and left inferior parietal lobule in ASD patients, indicating the possible role of the ACC in language communication (Kana et al., 2017). Our observation that the left ACC of *FMR1^−/−^* and VPA-pretreated mice lost responsiveness to USV indicated that stimulating the left ACC and associated brain regions would improve social communication in ASD patients.

One notable observation of our study is the alternation of syllable composition by modulating the left ACC neuronal activity. Previous studies have demonstrated that mouse USV syllables are plastic and scalable, depending on the identity of the environment or the reliability of the social context (Chen et al., 2021). Male mice emit simple syllables to signal courtship and complex syllables to lure fresh females (Chabout et al., 2015). Our data that both optogenetic manipulation and rTMS treatment increased the type 1 syllables (corresponding to the simple syllable) in *FMR1^−/−^* and VPA-pretreated mice indicated that stimulating the left ACC might enhance the active sociability of ASD mice.

Although optogenetic manipulation is very precise and efficient in animal studies, it is still a long way to clinical application. rTMS, due to its non-invasive property, has been successfully applied to mood-defective disorders (Perera et al., 2016). Previous animal studies, including ours, have demonstrated that rTMS modulation could improve social function (Tan et al., 2018; Hou et al., 2021). However, in most animal studies, the magnetic field covers the whole cortex, leaving the targeting area unspecific. Based on a two-coil TMS transducer that could adjust the orientation of the electric field rapidly and accurately (Souza et al., 2022), we developed a 3-coil TMS transducer that could confine the magnetic field within a size of 0.5–1.5mm^3^. For the first time, we applied this precise target TMS to a mouse study and validated the brain region specificity of this precise target rTMS by evaluating the left hindlimb movement and corresponding electromyographic activity. The improvement of USV communication in two ASD mouse models by left ACC modulation indicates that this brain region-focused rTMS could be expanded to other psychological disorders. Although the direct translation of the current study into the clinic is still challenging, the combination of TMS with functional neuroimaging such as magnetic resonance imaging may be a promising strategy in the future (Battaglia et al., 2023).

## Data availability statement

The original contributions presented in the study are included in the article/Supplementary material, further inquiries can be directed to the corresponding authors.

## Ethics statement

The animal study was approved by Animal Care and Use Committee of Fourth Military Medical University. The study was conducted in accordance with the local legislation and institutional requirements.

## Author contributions

YH: Visualization, Software, Methodology, Investigation, Formal analysis, Data curation, Writing – review & editing, Writing – original draft, Validation. YL: Writing – review & editing, Writing – original draft, Validation, Methodology, Investigation, Data curation. DY: Software, Formal analysis, Writing – review & editing, Writing – original draft, Investigation, Data curation. YZ: Methodology, Software, Investigation, Formal analysis, Data curation, Writing – review & editing. TF: Methodology, Investigation, Writing – review & editing. WZ: Methodology, Investigation, Writing – review & editing. PX: Methodology, Investigation, Writing – review & editing. XL: Validation, Supervision, Resources, Project administration, Conceptualization, Writing – review & editing, Writing – original draft. SW: Funding acquisition, Writing – review & editing, Writing – original draft, Supervision, Resources, Project administration, Conceptualization. YW: Validation, Writing – review & editing, Writing – original draft, Supervision, Resources, Project administration, Funding acquisition, Conceptualization.

## References

[ref1] Arriaga G. Jarvis E. D. (2013). Mouse vocal communication system: are ultrasounds learned or innate? Brain Lang. 124, 96–116. doi: 10.1016/j.bandl.2012.10.002, PMID: 23295209 PMC3886250

[ref2] Battaglia S. Schmidt A. Hassel S. Tanaka M. (2023). Editorial: case reports in neuroimaging and stimulation. Front. Psych. 14:1264669. doi: 10.3389/fpsyt.2023.1264669, PMID: 37599881 PMC10433894

[ref3] Block C. L. Eroglu O. Mague S. D. Smith C. J. Ceasrine A. M. Sriworarat C.. (2022). Prenatal environmental stressors impair postnatal microglia function and adult behavior in males. Cell Rep. 40:111161. doi: 10.1016/j.celrep.2022.111161, PMID: 35926455 PMC9438555

[ref4] Camacho-Conde J. A. Gonzalez-Bermudez M. D. R. Carretero-Rey M. Khan Z. U. (2022). Brain stimulation: a therapeutic approach for the treatment of neurological disorders. CNS Neurosci. Ther. 28, 5–18. doi: 10.1111/cns.13769, PMID: 34859593 PMC8673710

[ref5] Chabout J. Sarkar A. Dunson D. B. Jarvis E. D. (2015). Male mice song syntax depends on social contexts and influences female preferences. Front. Behav. Neurosci. 9:76. doi: 10.3389/fnbeh.2015.00076 25883559 PMC4383150

[ref6] Chen J. Markowitz J. E. Lilascharoen V. Taylor S. Sheurpukdi P. Keller J. A.. (2021). Flexible scaling and persistence of social vocal communication. Nature 593, 108–113. doi: 10.1038/s41586-021-03403-8, PMID: 33790464 PMC9153763

[ref7] Corballis M. C. (2014). Left brain, right brain: facts and fantasies. PLoS Biol. 12:e1001767. doi: 10.1371/journal.pbio.1001767, PMID: 24465175 PMC3897366

[ref8] Dai Y. Liu Y. Zhang L. Ren T. Wang H. Yu J.. (2022). Shanghai autism early development: an integrative Chinese ASD cohort. Neurosci. Bull. 38, 1603–1607. doi: 10.1007/s12264-022-00904-y, PMID: 35739378 PMC9723093

[ref9] Deemyad T. (2022). Lateralized changes in language associated auditory and somatosensory cortices in autism. Front. Syst. Neurosci. 16:787448. doi: 10.3389/fnsys.2022.787448, PMID: 35300070 PMC8923120

[ref10] Ehret G. (1987). Left hemisphere advantage in the mouse brain for recognizing ultrasonic communication calls. Nature 325, 249–251. doi: 10.1038/325249a0, PMID: 3808021

[ref11] El-Gaby M. Shipton O. A. Paulsen O. (2015). Synaptic plasticity and memory: new insights from hippocampal left-right asymmetries. Neuroscientist 21, 490–502. doi: 10.1177/1073858414550658 25239943

[ref12] Gan-Or B. London M. (2023). Cortical circuits modulate mouse social vocalizations. Sci. Adv. 9:eade6992. doi: 10.1126/sciadv.ade6992, PMID: 37774030 PMC10541007

[ref13] Gao S. C. Wei Y. C. Wang S. R. Xu X. H. (2019). Medial preoptic area modulates courtship ultrasonic vocalization in adult male mice. Neurosci. Bull. 35, 697–708. doi: 10.1007/s12264-019-00365-w, PMID: 30900143 PMC6616611

[ref14] Guo B. Chen J. Chen Q. Ren K. Feng D. Mao H.. (2019). Anterior cingulate cortex dysfunction underlies social deficits in shank 3 mutant mice. Nat. Neurosci. 22, 1223–1234. doi: 10.1038/s41593-019-0445-9, PMID: 31332372

[ref15] Hamed A. Daszczuk P. Kursa M. B. Turzynska D. Sobolewska A. Lehner M.. (2016). Non-parametric analysis of neurochemical effects and arc expression in amphetamine-induced 50-kHz ultrasonic vocalization. Behav. Brain Res. 312, 174–185. doi: 10.1016/j.bbr.2016.05.042, PMID: 27288591

[ref16] Hodges S. L. Nolan S. O. Reynolds C. D. Lugo J. N. (2017). Spectral and temporal properties of calls reveal deficits in ultrasonic vocalizations of adult Fmr 1 knockout mice. Behav. Brain Res. 332, 50–58. doi: 10.1016/j.bbr.2017.05.052, PMID: 28552599 PMC6503674

[ref17] Hou Y. Zhao J. Yang D. Xuan R. Xie R. Wang M.. (2021). LF-rTMS ameliorates social dysfunction of FMR1(−/−) mice via modulating Akt/GSK-3beta signaling. Biochem. Biophys. Res. Commun. 550, 22–29. doi: 10.1016/j.bbrc.2021.02.086, PMID: 33677132

[ref18] Kana R. K. Sartin E. B. Stevens C. Jr. Deshpande H. D. Klein C. Klinger M. R.. (2017). Neural networks underlying language and social cognition during self-other processing in autism spectrum disorders. Neuropsychologia 102, 116–123. doi: 10.1016/j.neuropsychologia.2017.06.008, PMID: 28619530

[ref19] Kim C. K. Adhikari A. Deisseroth K. (2017). Integration of optogenetics with complementary methodologies in systems neuroscience. Nat. Rev. Neurosci. 18, 222–235. doi: 10.1038/nrn.2017.15, PMID: 28303019 PMC5708544

[ref20] Lahvis G. P. Alleva E. Scattoni M. L. (2011). Translating mouse vocalizations: prosody and frequency modulation. Genes Brain Behav. 10, 4–16. doi: 10.1111/j.1601-183X.2010.00603.x, PMID: 20497235 PMC2936813

[ref21] Lord C. Elsabbagh M. Baird G. Veenstra-Vanderweele J. (2018). Autism spectrum disorder. Lancet 392, 508–520. doi: 10.1016/S0140-6736(18)31129-2, PMID: 30078460 PMC7398158

[ref22] Lundstrom S. Taylor M. Larsson H. Lichtenstein P. Kuja-Halkola R. Gillberg C. (2022). Perceived child impairment and the 'autism epidemic'. J. Child Psychol. Psychiatry 63, 591–598. doi: 10.1111/jcpp.13497, PMID: 34363395

[ref23] Perera T. George M. S. Grammer G. Janicak P. G. Pascual-Leone A. Wirecki T. S. (2016). The clinical TMS Society consensus review and treatment recommendations for TMS therapy for major depressive disorder. Brain Stimul. 9, 336–346. doi: 10.1016/j.brs.2016.03.010, PMID: 27090022 PMC5612370

[ref24] Pietropaolo S. Crusio W. E. D'amato F. (2017). Treatment approaches in rodent models for autism Spectrum disorder. Curr. Top. Behav. Neurosci. 30, 325–340. doi: 10.1007/7854_2015_433 26857461

[ref25] Reindal L. Naerland T. Weidle B. Lydersen S. Andreassen O. A. Sund A. M. (2023). Structural and pragmatic language impairments in children evaluated for autism Spectrum disorder (ASD). J. Autism Dev. Disord. 53, 701–719. doi: 10.1007/s10803-020-04853-1, PMID: 33515169 PMC9944009

[ref26] Rolls E. T. (2019). The cingulate cortex and limbic systems for emotion, action, and memory. Brain Struct. Funct. 224, 3001–3018. doi: 10.1007/s00429-019-01945-2, PMID: 31451898 PMC6875144

[ref27] Saturnino G. B. Puonti O. Nielsen J. D. Antonenko D. Madsen K. H. Thielscher A. (2019). “SimNIBS 2.1: a comprehensive pipeline for individualized electric field modelling for transcranial brain stimulation” in Brain and human body modeling: Computational human modeling at EMBC 2018. eds. Makarov S. Horner M. Noetscher G. (Cham: Springer), 3–25. 31725247

[ref28] Saturnino G. B. Wartman W. A. Makarov S. N. Thielscher A. (2020). Accurate TMS head modeling: interfacing SimNIBS and BEM-FMM in a MATLAB-based module. Annu. Int. Conf. IEEE Eng. Med. Biol. Soc. 2020, 5326–5329. doi: 10.1109/EMBC44109.2020.9175802, PMID: 33019186

[ref29] Schneider K. N. Sciarillo X. A. Nudelman J. L. Cheer J. F. Roesch M. R. (2020). Anterior cingulate cortex signals attention in a social paradigm that manipulates reward and shock. Curr. Biol. 30:e3722, 3724–3735.e2. doi: 10.1016/j.cub.2020.07.039 PMC7541607 32763169

[ref30] Schroeder J. C. Reim D. Boeckers T. M. Schmeisser M. J. (2017). Genetic animal models for autism Spectrum disorder. Curr. Top. Behav. Neurosci. 30, 311–324. doi: 10.1007/7854_2015_407, PMID: 26602248

[ref31] Silleresi S. Prevost P. Zebib R. Bonnet-Brilhault F. Conte D. Tuller L. (2020). Identifying language and cognitive profiles in children with ASD via a cluster analysis exploration: implications for the new ICD-11. Autism Res. 13, 1155–1167. doi: 10.1002/aur.2268 31985169

[ref32] Souza V. H. Nieminen J. O. Tugin S. Koponen L. M. Baffa O. Ilmoniemi R. J. (2022). TMS with fast and accurate electronic control: measuring the orientation sensitivity of corticomotor pathways. Brain Stimul. 15, 306–315. doi: 10.1016/j.brs.2022.01.009, PMID: 35038592

[ref33] Sungur A. O. Schwarting R. K. Wohr M. (2016). Early communication deficits in the shank 1 knockout mouse model for autism spectrum disorder: developmental aspects and effects of social context. Autism Res. 9, 696–709. doi: 10.1002/aur.1564, PMID: 26419918

[ref34] Takumi T. Tamada K. Hatanaka F. Nakai N. Bolton P. F. (2020). Behavioral neuroscience of autism. Neurosci. Biobehav. Rev. 110, 60–76. doi: 10.1016/j.neubiorev.2019.04.012 31059731

[ref35] Tan T. Wang W. Xu H. Huang Z. Wang Y. T. Dong Z. (2018). Low-frequency rTMS ameliorates autistic-like behaviors in rats induced by neonatal isolation through regulating the synaptic GABA transmission. Front. Cell. Neurosci. 12:46. doi: 10.3389/fncel.2018.00046, PMID: 29541022 PMC5835518

[ref36] Tasaka G. I. Guenthner C. J. Shalev A. Gilday O. Luo L. Mizrahi A. (2018). Genetic tagging of active neurons in auditory cortex reveals maternal plasticity of coding ultrasonic vocalizations. Nat. Commun. 9:871. doi: 10.1038/s41467-018-03183-2, PMID: 29491360 PMC5830453

[ref37] Tervo D. G. R. Proskurin M. Manakov M. Kabra M. Vollmer A. Branson K.. (2014). Behavioral variability through stochastic choice and its gating by anterior cingulate cortex. Cell 159, 21–32. doi: 10.1016/j.cell.2014.08.037, PMID: 25259917

[ref38] Tschida K. Michael V. Takatoh J. Han B. X. Zhao S. Sakurai K.. (2019). A specialized neural circuit gates social vocalizations in the mouse. Neuron 103:e454, 459–472.e4. doi: 10.1016/j.neuron.2019.05.025 PMC6687542 31204083

[ref39] Tsuji T. Mizutani R. Minami K. Furuhara K. Fujisaku T. Pinyue F.. (2021). Oxytocin administration modulates the complex type of ultrasonic vocalisation of mice pups prenatally exposed to valproic acid. Neurosci. Lett. 758:135985. doi: 10.1016/j.neulet.2021.135985, PMID: 34048819

[ref40] Von Merten S. Hoier S. Pfeifle C. Tautz D. (2014). A role for ultrasonic vocalisation in social communication and divergence of natural populations of the house mouse (*Mus musculus domesticus*). PLoS One 9:e97244. doi: 10.1371/journal.pone.0097244, PMID: 24816836 PMC4016290

