# Supplementary material for: Involvement and regulation of the left anterior cingulate cortex in the ultrasonic communication deficits of autistic mice
Source: Front Behav Neurosci. 2024 May 15;18:1387447. doi: 10.3389/fnbeh.2024.1387447 (PMC11133516; doi:10.3389/fnbeh.2024.1387447)
Supplement: Supplementary file 1 [file Data_Sheet_1.docx]

Supplementary Material

Involvement and regulation of left anterior cingulate cortex in the ultrasonic communication deficiency of autistic mice

Yilin Hou^1#^, Yuqian Li^2#^, Dingding Yang^2#^, Youyi Zhao^3^, Tingwei Feng^1^, Wei’an Zheng^1^, Panpan Xian^1^, Xufeng Liu^1^*, Shengxi Wu^2^*, Yazhou Wang^2^*

# Supplementary Figures

#
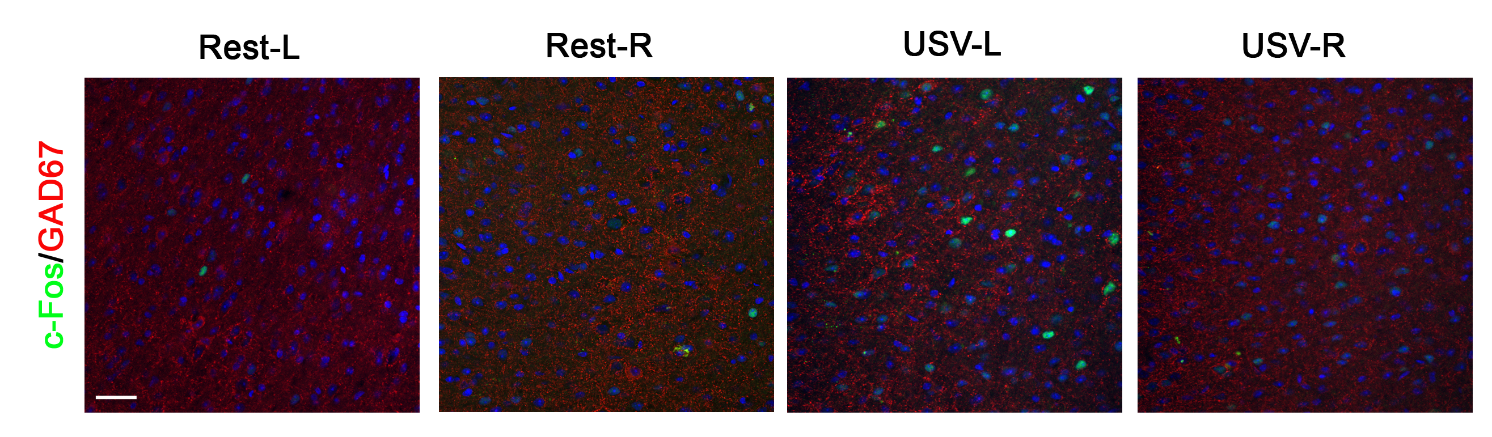


**Supplementary Figure 1.** Double-immunostaining of c-Fos/GAD67 in the left (L) and right (R) ACC of WT mice under normal condition (at rest) and following USV induction.


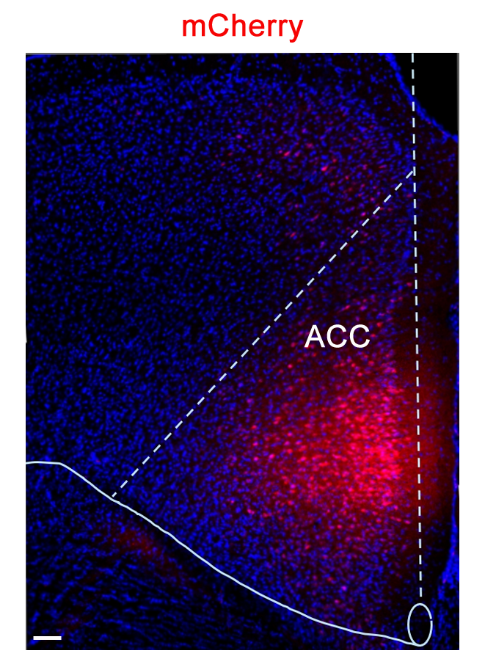


**Supplementary Figure 2.** Verification of ACC injection and virus infection by the expression of mCherry. Bar = 100 μm.


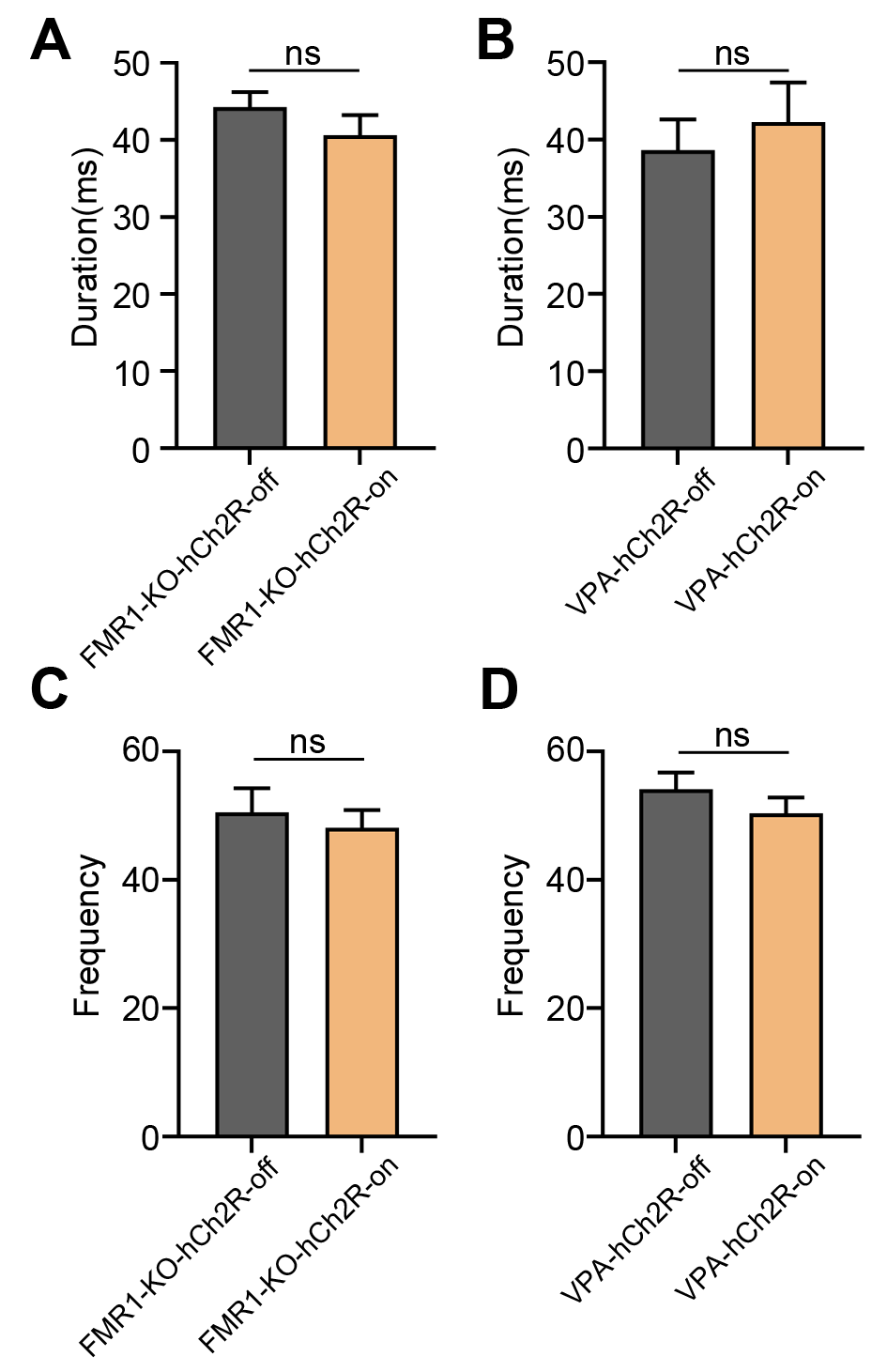


**Supplementary Figure 3.** Effects of optogenetic activating left ACC neurons on the USV duration and frequency of *FMR1^-/-^* (FMR1-KO) mice and VPA-pretreated mice.


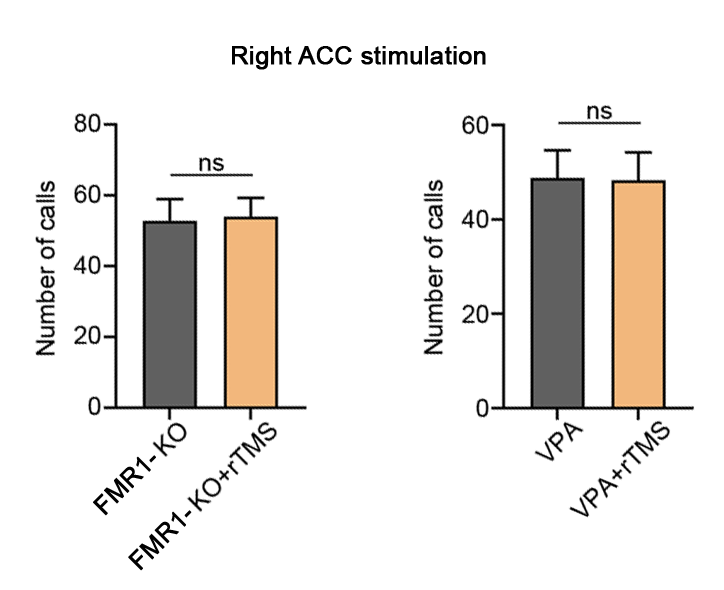


**Supplementary Figure 4.** Effects of right ACC modulation on USV calls of *FMR1^-/-^* (FMR1-KO) mice and VPA-induced ASD mice.

**Supplementary video.** Supplementary video corresponding to supplementary figure 2 showing the movement of left hindlimb upon TMS stimulation of right motor cortex.
